# A survival nomogram model constructed with common clinical characteristics to assist clinical decisions for diffuse low-grade gliomas: A population analysis based on SEER database

**DOI:** 10.3389/fonc.2023.963688

**Published:** 2023-02-09

**Authors:** Lei Ao, Dongjie Shi, Dan Liu, Hua Yu, Li Xu, Yongzhi Xia, Shilei Hao, Yaying Yang, Wenjie Zhong, Junjie Zhou, Haijian Xia

**Affiliations:** ^1^ Department of Neurosurgery, the First Affiliated Hospital of Chongqing Medical University, Chongqing, China; ^2^ Health Management Center, The First Affiliated Hospital of Chongqing Medical University, Chongqing, China; ^3^ Key Laboratory of Biorheological Science and Technology, Ministry of Education, College of Bioengineering, Chongqing University, Chongqing, China; ^4^ Department of Pathology, Molecular Medicine and Tumor Center, Chongqing Medical University, Chongqing, China

**Keywords:** diffuse low-grade gliomas, nomogram, prognostic model, SEER database, overall survival

## Abstract

**Background:**

The prognosis of diffuse low-grade gliomas (DLGGs, WHO grade 2) is highly variable, making it difficult to evaluate individual patient outcomes. In this study, we used common clinical characteristics to construct a predictive model with multiple indicators.

**Methods:**

We identified 2459 patients diagnosed with astrocytoma and oligodendroglioma from 2000 to 2018 in the SEER database. After removing invalid information, we randomly divided the cleaned patient data into training and validation groups. We performed univariate and multivariate Cox regression analyses and constructed a nomogram. Receiver operating characteristic (ROC) curve, c-index, calibration curve, and subgroup analyses were used to assess the accuracy of the nomogram by internal and external validation.

**Results:**

After univariate and multivariate Cox regression analyses, we identified seven independent prognostic factors, namely, age (*P<0.001*), sex (*P<0.05*), histological type (*P<0.001*), surgery (*P<0.01*), radiotherapy (*P<0.001*), chemotherapy (*P<0.05*) and tumor size (*P<0.001*). The ROC curve, c-index, calibration curve, and subgroup analyses of the training group and the validation group showed that the model had good predictive value. The nomogram for DLGGs predicted patients’ 3-, 5- and 10-year survival rates based on these seven variables.

**Conclusions:**

The nomogram constructed with common clinical characteristics has good prognostic value for patients with DLGGs and can help physicians make clinical decisions.

## 1 Introduction

Diffuse low-grade gliomas (DLGGs), including astrocytomas and oligodendrogliomas (World Health Organization [WHO] grade 2), show diverse biological characteristics and variable clinical behaviors (1, 2). DLGGs often recur after surgery and have the potential to transform into higher-grade gliomas, which often indicates poor prognosis for patients (2, 3).

The treatment of patients with DLGGs generally includes observation, surgery, radiotherapy, and chemotherapy (4, 5). Surgical resection, as the primary and most important treatment, is closely related to the prognosis of patients with DLGGs (6). The guidelines for glioma recommend that surgical treatment strategies for patients with DLGGs include total resection or even supratotal resection of tumors in nonfunctional areas (7). Maximized safe resection can reduce the probability of tumor recurrence after surgery (8). DLGGs are sensitive to chemotherapy, and the chemotherapy regimens for DLGGs generally include procarbazine, lomustine, and vincristine (PCV) and temozolomide (TMZ) (9). However, there is no consensus on a strategy for the dose of radiotherapy (9, 10). Therefore, it is necessary to explore the impact of these treatment strategies on the prognosis of patients with DLGGs.

Currently, molecular markers used for identifying and classifying gliomas, including isocitrate dehydrogenase 1 (IDH1) mutation, O6-methylguanylmethyltransferase (MGMT) promoter methylation, 1p/19q chromosome deletion and p53 mutation, have also been shown to have prognostic value for patients with DLGGs (11–16). The new 2021 WHO classification guidelines indicate that the combination of molecular markers and histological features provides more accurate clinical stratification of DLGG patients (17).

In addition to molecular markers, emerging studies have demonstrated that clinical characteristics show good outcome prediction performance for patients with lower-grade gliomas (11, 13). It has been reported that age > 50, tumor size > 4 cm, tumor location, and a Karnofsky performance status (KPS) score less than 80 are risk factors for a shorter overall survival (OS) in patients with lower-grade gliomas (18). Since these characteristics are all easy to assess in the clinic, the predictive performance of a combination of these characteristics for patient survival have great potential (17). Thus, we aimed to develop a predictive framework including multiple clinical risk factors to evaluate the survival performance of patients with DLGGs.

Patients with DLGGs usually have a long follow-up time, making it difficult to collect enough samples from separate medical centers (7, 17). Therefore, we obtained relevant data from the Surveillance, Epidemiology, and End Results (SEER) database, which contains demographic and clinical information about patients with various cancers in the USA (19). Regarding methodology, nomograms have been used in the prognostication of various tumors, such as breast cancer (20), bladder cancer (21), and lung cancer (22), since they intuitively describe the statistical results graphically (23). However, there is still a lack of prognostic models for DLGGs. A nomogram was constructed in this study from common clinical characteristics to predict the outcome of patients with DLGGs, and it was validated internally and externally to ensure that it had good predictive power.

## 2 Patients and methods

### 2.1 Patients

Molecular reclassification of the DLGGs following the WHO 2021 criteria is based on IDH mutation status, 1p/19q codeletion status, and histopathological grade (17). The new criteria classify poorly defined entities into more objectively defined types according to molecular characteristics; for example, oligoastrocytoma is classified as astrocytoma or oligodendroglioma (17). Considering the lack of molecular markers in the SEER database, to minimize the heterogeneity of enrolled cases and maximize the reflection of the real situation based on the 2021 WHO classification system, patients with astrocytoma (WHO grade 2) and oligodendroglioma (WHO grade 2) were enrolled as DLGG cases in the current study.

We identified 2459 cases of astrocytoma and oligodendroglioma between 2000 and 2018 in the SEER database (version 8.3.9); the data we obtained included the survival status, survival time, age, sex, year of diagnosis, race, histological type, laterality, surgery, radiotherapy, chemotherapy, and tumor size. Then, we used Excel (version 2019) to remove invalid information containing blank values.

### 2.2 Statistical analysis

#### 2.2.1 Univariate and multivariate Cox regression analysis

We used RStudio (version 2021.09.2-382) to randomly divide the patients into the training group and the validation group at a ratio of 7:3. The data in the training group were used to verify the independent prognostic factors for OS and construct the nomogram. The data in the validation group were used to verify the reliability of the results obtained from the training group. Cox regression is a widely used statistical method, and it is often used to analyze the effect of multiple risk factors on survival (24). Therefore, this study applied univariate and multivariate Cox regression analyses to determine the impact of independent prognostic factors on the OS of patients with DLGGs. In the training group, we used the “rms”, “foreign” and “survival” packages to perform univariate Cox regression and identify factors correlated with the outcomes of patients with DLGGs. Then, the significant factors obtained in the univariate Cox regression were analyzed by multivariate Cox regression to determine the independent prognostic factors. We calculated the P values, hazard ratios (HRs), and 95% confidence intervals (CIs) of the independent prognostic factors. A P value less than 0.05 was regarded as statistically significant.

#### 2.2.2 Construction of the nomogram

In the multivariate Cox regression analysis, these independent prognostic factors were assigned different regression coefficients according to their contribution to the outcome variable. Each patient’s score was calculated by the value level of these independent factors, and then the score was added to obtain the total score. Finally, the prognostic value of the individual outcome event was calculated through the functional transformation between the total score and the probability of the outcome event. This prognostic value indicates the survival prognosis of patients based on the nomogram. We visualized the survival rates at 3 years, 5 years, and 10 years for patients with DLGGs.

#### 2.2.3 Kaplan–Meier survival curve and subgroup analyses

We used Cox regression to select independent prognostic factors for the OS of patients with DLGGs. The “survival” package was used to score the patients in the training group according to these factors. We divided the DLGG patients into a high-risk group and a low-risk group by the median risk score. Then, we used the “survival” package to draw the Kaplan–Meier survival curve of the high-risk group and the low-risk group. According to the independent prognostic factors, the training group was divided into different subgroups. Through subgroup analysis, we evaluated the effectiveness of our prognostic model in each subgroup.

### 2.3 Internal validation including the ROC curve, c-index, and calibration curve

We used the “survivalROC” package to draw 3-year, 5-year, and 10-year ROC curves of patients with DLGGs to evaluate the accuracy of the model. The area under the ROC curve (AUC) and c-index can show the accuracy of the model. The closer the values of the AUC and the c-index are to 1, the better the authenticity of the model (25). Thus, we also calculated the c-index and drew a calibration curve to further verify the predictive value of the nomogram.

### 2.4 External validation

We performed external validation by the validation group. Based on the median derived from the training group, the validation group was divided into a high-risk group and a low-risk group. We drew Kaplan–Meier survival curves for the high-risk group and the low-risk group. We also performed subgroup analysis in the validation group. Then, we calculated the c-index of the validation group and drew the ROC curve and the validation curve to verify the accuracy of the model.

## 3 Results

### 3.1 Patient characteristics

**Figure 1:**
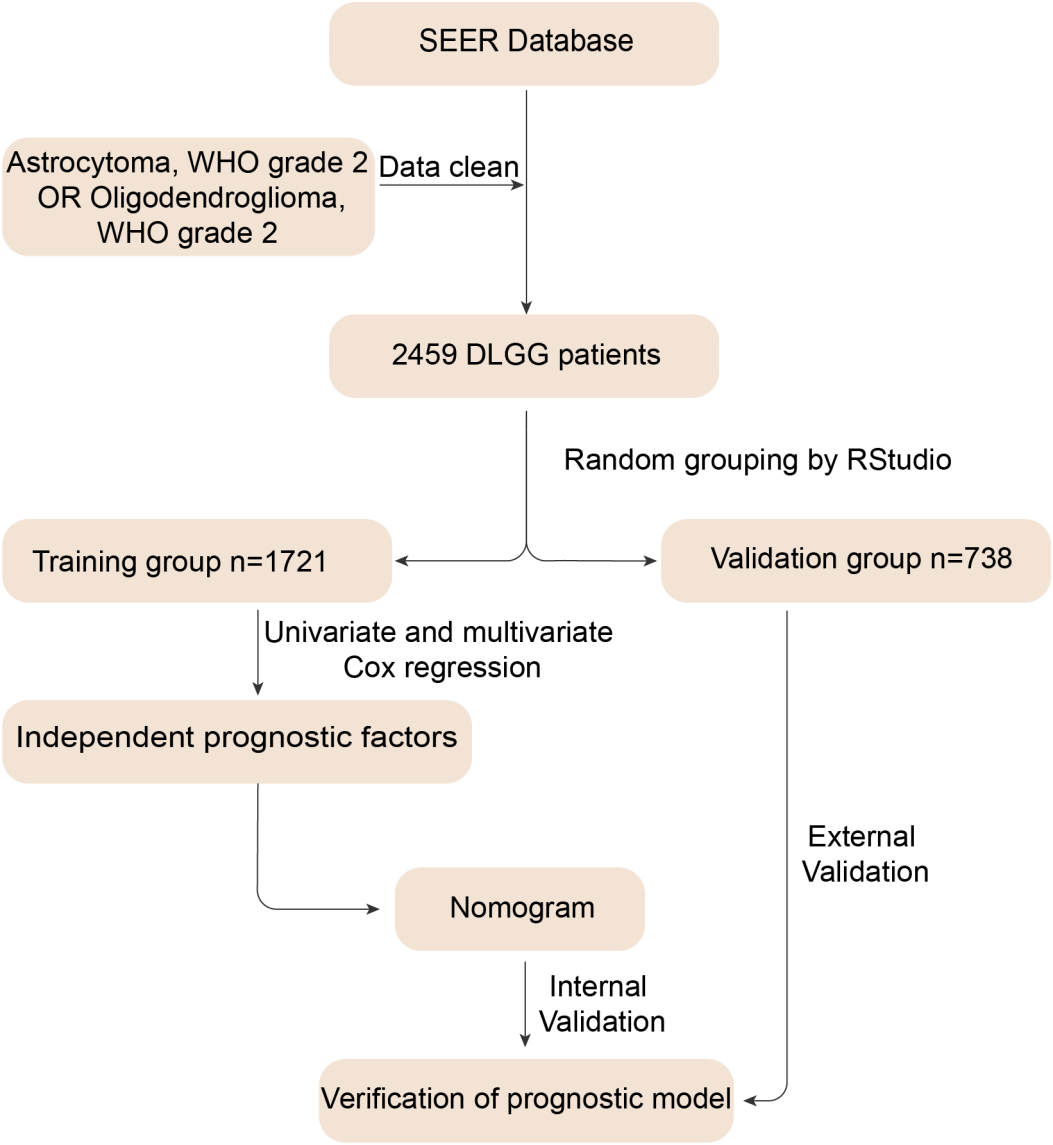
Flow chart for constructing the nomogram. First, we identified 2459 patients diagnosed with astrocytoma and oligodendroglioma and removed invalid information. Then, we randomly divided the data into training and validation groups. The training group data were used to derive independent prognostic factors and construct a nomogram. Finally, we performed internal and external validation to verify the predictive value of the nomogram.

**Figure 1** shows the flow chart of the study. After screening, 2459 DLGG patients who met the criteria were enrolled in this study (**Supplementary Table 1**). The clinical characteristics of the enrolled patients, including age, sex, etc., are presented in **Table 1**. Next, we randomized all patients into a training group (1721 patients) and a validation group (738 patients). As presented in **Table 1**, women accounted for 42.18% and men accounted for 57.82% of the patients in the training group, similar to the ratio in the validation group (women: 44.85%, men: 55.15%). For the other clinical characteristics, there were no obvious differences observed between the training and validation groups (**Table 1**).

**Table 1:** The clinical characteristics of the enrolled patients.

Variable	Total population	After cleaning	
		Training group Cases (%)	Validation group Cases (%)
Age			
0-44	1489	1048 (60.89%)	441 (59.76%)
45-59	599	417 (24.23%)	182 (24.66%)
60-74	292	199 (11.56%)	93 (12.60%)
≥75	79	57 (3.31%)	22 (2.98%)
Sex			
Female	1057	726 (42.18%)	331 (44.85%)
Male	1402	995 (57.82%)	407 (55.15%)
Year of diagnosis			
2000-2005	90	63 (3.66%)	27 (3.66%)
2006-2008	201	143 (8.31%)	58 (7.86%)
2009-2012	712	495 (28.76%)	217 (29.40%)
2013-2017	1456	1020 (59.27%)	436 (59.08%)
Race			
White	2130	1490 (86.58%)	640 (86.72%)
Black	171	122 (7.09%)	49 (6.64%)
Asian or pacific islander	135	96 (5.58%)	39 (5.28%)
American Indian	23	13 (0.76%)	10 (1.36%)
Histological type			
Astrocytoma, WHO grade 2	1258	868 (50.44%)	390 (52.85%)
Oligodendroglioma, WHO grade 2	1201	853 (49.56%)	348 (47.15%)
Laterality			
Not a paired site	221	150 (8.72%)	71 (9.62%)
Left	1074	759 (44.10%)	315 (42.68%)
Right	1141	800 (46.48%)	341 (46.21%)
Other	23	12 (0.70%)	11 (1.49%)
Surgery			
Biopsy	807	567 (32.95%)	240 (32.52%)
STR	703	482 (28.01%)	221 (29.95%)
GTR	949	672 (39.05%)	277 (37.53%)
Radiotherapy			
No	1460	1011 (58.74%)	449 (60.84%)
Yes	999	710 (41.26%)	289 (39.16%)
Chemotherapy			
No	1526	1072 (62.29%)	454 (61.52%)
Yes	933	649 (37.71%)	284 (38.48%)
Tumor size(mm)			
0-39	1087	749 (43.52%)	338 (45.80%)
40-59	834	587 (34.11%)	247 (33.47%)
≥60	538	385 (22.37%)	153 (20.73%)

For “Laterality”, the variable “not a paired site” means the tumor has no obvious laterality. The variable “Other” includes that the tumor only involves one side, but the right or left side is not specified; or that both sides are involved at the time of diagnosis, but the unilateral origin is unknown.

For “Surgery”, “STR” represents “subtotal resection of tumor”, and “GTR” represents “gross total resection of tumor”.

### 3.2 Establishment of the prognostic model

**Figure 2:**
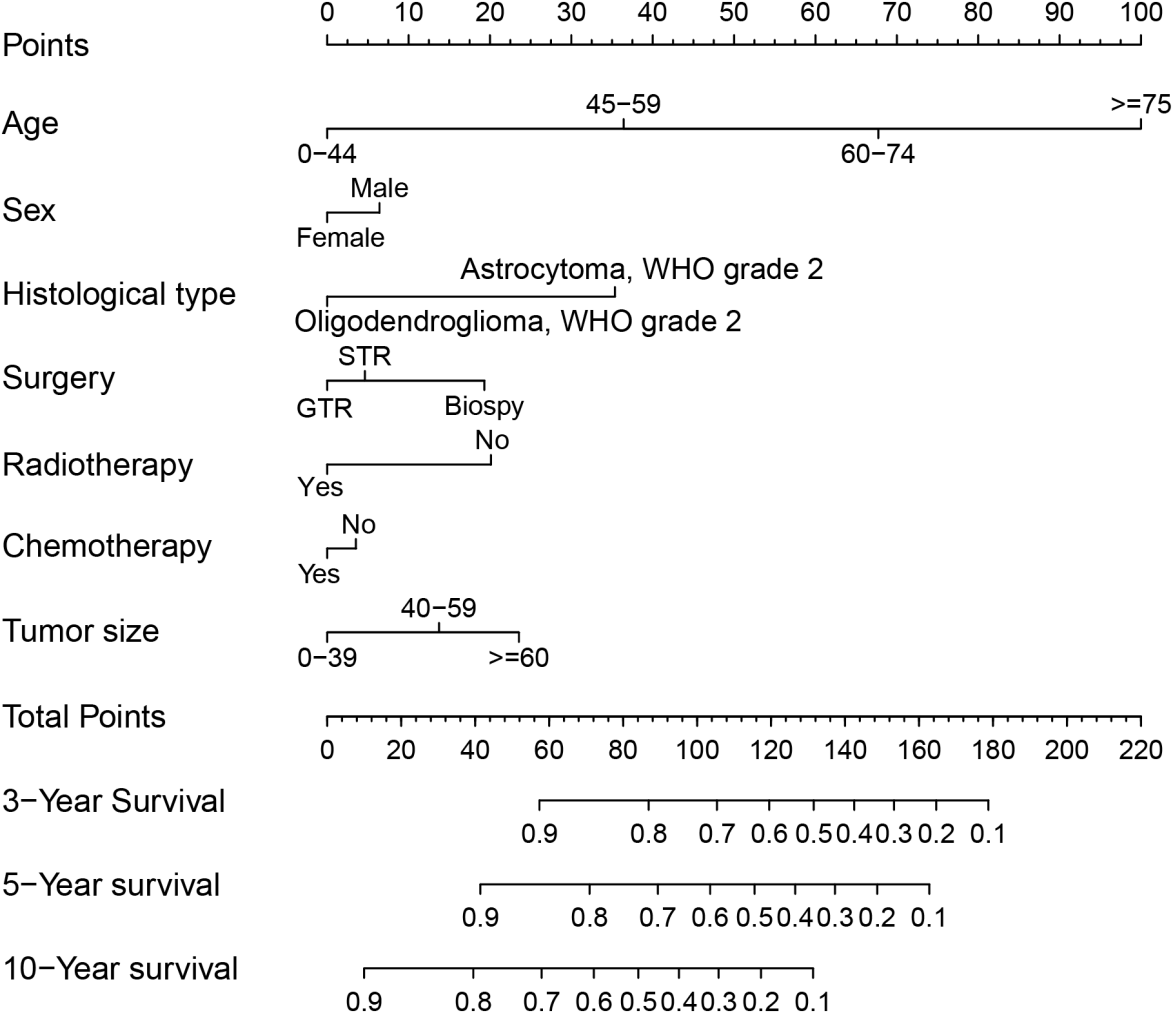
Nomogram for prognostication of patients with DLGGs. Independent prognostic factors included age *(P<0.001)*, sex *(P<0.05)*, histological type *(P<0.001)*, surgery *(P<0.001)*, radiotherapy *(P<0.001)*, chemotherapy *(P<0.05)*, and tumor size *(P<0.001)*. A vertical line is drawn upward, and the intersection with the point-line is the score for each variable. We calculated the total score for each patient and predicted 3-year, 5-year, and 10-year OS. “STR”, “subtotal resection of tumor”; “GTR”, “gross total resection of tumor”.

**Figure 3:**
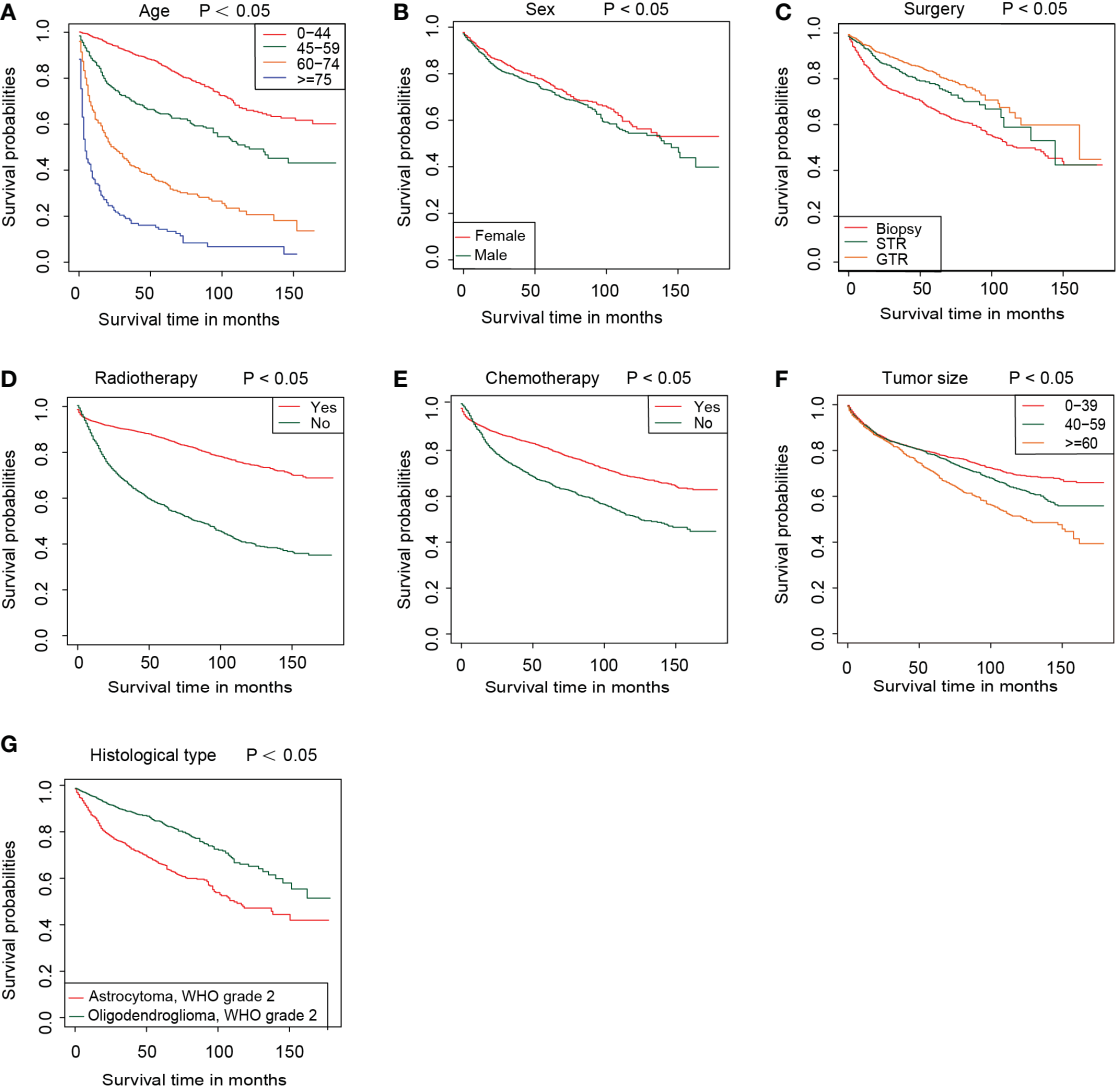
The survival curve in different subgroups, including age **(A)**, sex **(B)**, surgery **(C)**, radiotherapy **(D)**, chemotherapy **(E)**, tumor size **(F)**, and histological type **(G)** in the training group.

First, we used univariate Cox regression analysis to screen out the clinical characteristics. As shown in **Table 2**, age (*P<0.001*), sex (*P<0.01*), histological type (*P<0.001*), laterality (*P<0.05*), surgery (*P<0.001*), radiotherapy (*P<0.001*), chemotherapy (*P<0.001*) and tumor size (*P<0.05*) were significantly correlated with patient prognosis. After multivariate Cox regression, we verified that age (*P<0.001*), sex (*P<0.05*), histological type (*P<0.001*), surgery (*P<0.001*), radiotherapy (*P<0.001*), chemotherapy (*P<0.05*) and tumor size (*P<0.001*) were independent prognostic factors for OS (**Table 2**). Based on these independent prognostic factors, we constructed a nomogram to predict the patient outcome, as shown in **Figure 2**. We generated prognostic curves for these factors (**Figures 3A–G**).

**Table 2:** Univariate and multivariate Cox regression analyses of the training group.

Variable	Univariate Cox regression			Multivariate Cox regression		
	HR	95% CI	P values	HR	95% CI	P values
Age						
0-44	-	-	-	-	-	-
45-59	2.3290	0.8455-2.3292	< 2e-16***	2.5242	2.0495-3.1088	< 2e-16***
60-74	5.5760	1.7184-5.5756	< 2e-16***	5.5916	4.4968-6.9529	< 2e-16***
≥75	12.6060	2.5341-12.6056	< 2e-16***	12.6636	9.3591-17.1348	< 2e-16***
Sex						
Female	–	–	–	–	–	–
Male	1.1780	1.1992-1. 5890	0.00255**	1.1774	1.0974-1.3897	0.036673*
Year of diagnosis						
2000-2005	-	-	-			
2006-2008	0.9316	0.8554-1.1380	0.6762			
2009-2012	0.8346	0.6911-1.190	0.4370			
2013-2017	0.7934	0.7733-1.2330	0.4170			
Race						
White	–	–	–			
Black	0.7769	0.5662-1.311	0.2380			
Asian or pacific islander	0.9129	0.6592-1.383	0.7870			
American Indian	0.8431	0.2906-2.1105	0.8415			
Histological type						
Astrocytoma, WHO grade 2	-	-	-			
Oligodendroglioma, WHO grade 2	0.4618	0.3784-0.5075	<2e-16***	0.4072	0.3411-0.4862	< 2e-16***
Laterality						
Not a paired site	-	-	-	-	-	-
Left	1.3320	1.0155-1.5570	0.03579*	1.0042	0.8264-1.1585	0.821223
Right	1.4120	1.0928-1.5596	0.02532*	1.0201	0.8138-1.1767	0.896019
Other	1.7014	0.9113-2.3420	0.06556	1.0332	0.9246-1.2995	0.733318
Surgery						
Biopsy	–	–	–	–	–	–
STR	0.6598	0.5357-0.8126	9.15e-05***	0.6885	0.5579-0.8498	0.00051***
GTR	0.4988	0.4075-0.6107	1.60e-11***	0.6121	0.4992-0.7505	2.36e-06***
Radiotherapy						
No	-	-	-	-	-	-
Yes	0.5101	0.5101-0.7846	4.23e-06***	0.5996	0.4221-0.7326	1.19e-07***
Chemotherapy						
No	–	–	–	–	–	–
Yes	0.5910	0.5590-0.7911	3.31e-08***	0.9144	0.8281-0.9181	0.021564*
Tumor size(mm)						
0-39	-	-	-	-	-	-
40-59	1.1800	1.0280-1.4240	0.0382*	1.4177	1.1685-1.7201	0.000402***
≥60	1.5080	1.2280-1.8500	7.51e-05***	1.8198	1.4694-2.2539	4.11e-08***

For “Laterality”, the variable “not a paired site” means the tumor has no obvious laterality. For “Surgery”, “STR” represents “subtotal resection of tumor”, and “GTR” represents “gross total resection of tumor”. The individual variables for multivariate Cox regression analysis included age, sex, histological type, laterality, surgery, radiotherapy, chemotherapy, and tumor size. Independent prognostic factors used to construct the nomogram included age, sex, histological type, surgery, radiotherapy, chemotherapy, and tumor size.*P <0.05;**P < 0.01;***P < 0.001.

### 3.3 Kaplan–Meier survival curve and subgroup analyses

**Figure 4:**
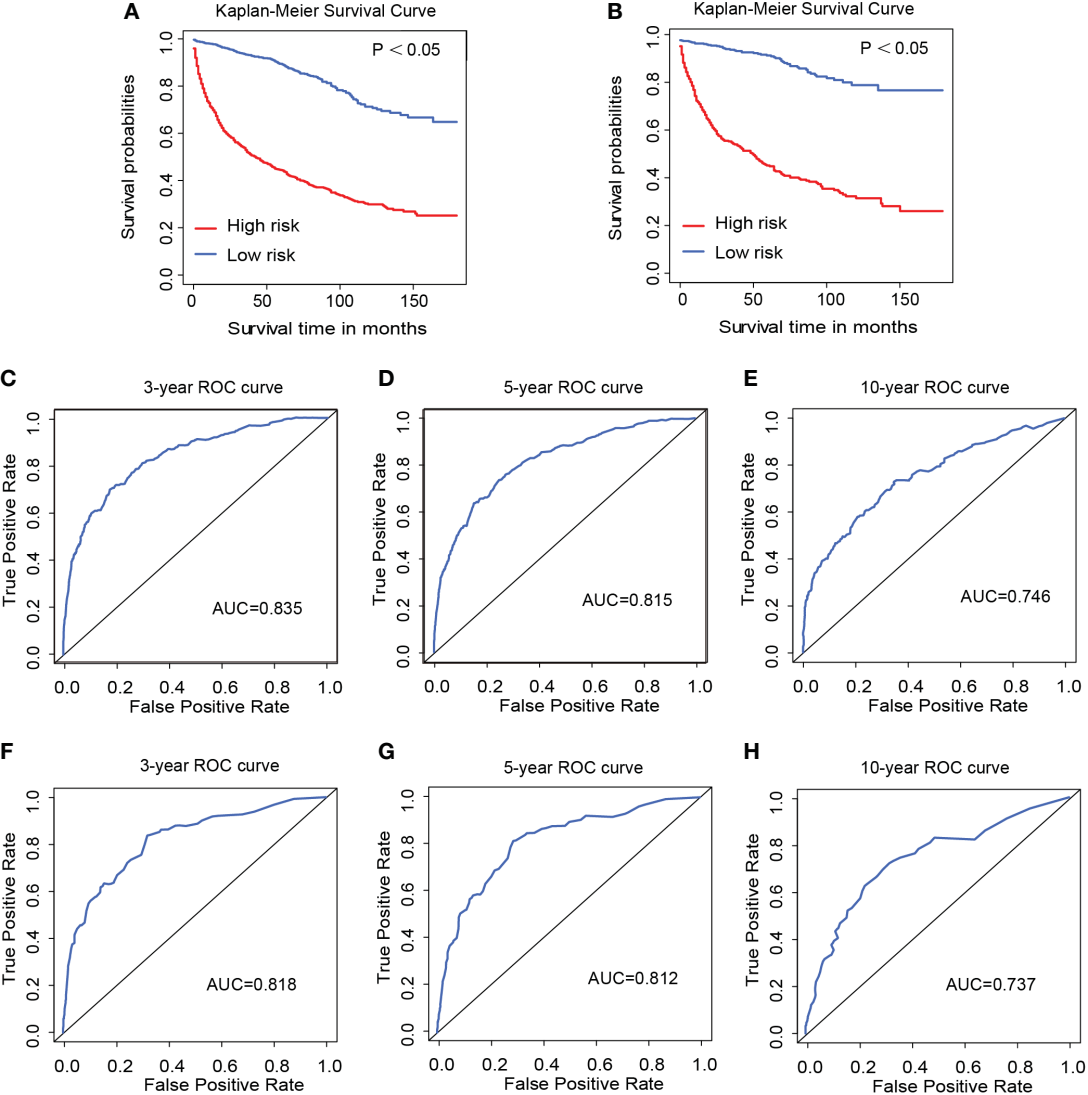
Kaplan–Meier survival curves of the training group **(A)** and validation group **(B)**. The 3-year **(C)**, 5-year **(D)**, and 10-year **(E)** ROC curves of the training group and the 3-year **(F)**, 5-year **(G)**, and 10-year **(H)** ROC curves of the validation group.

**Figure 5:**
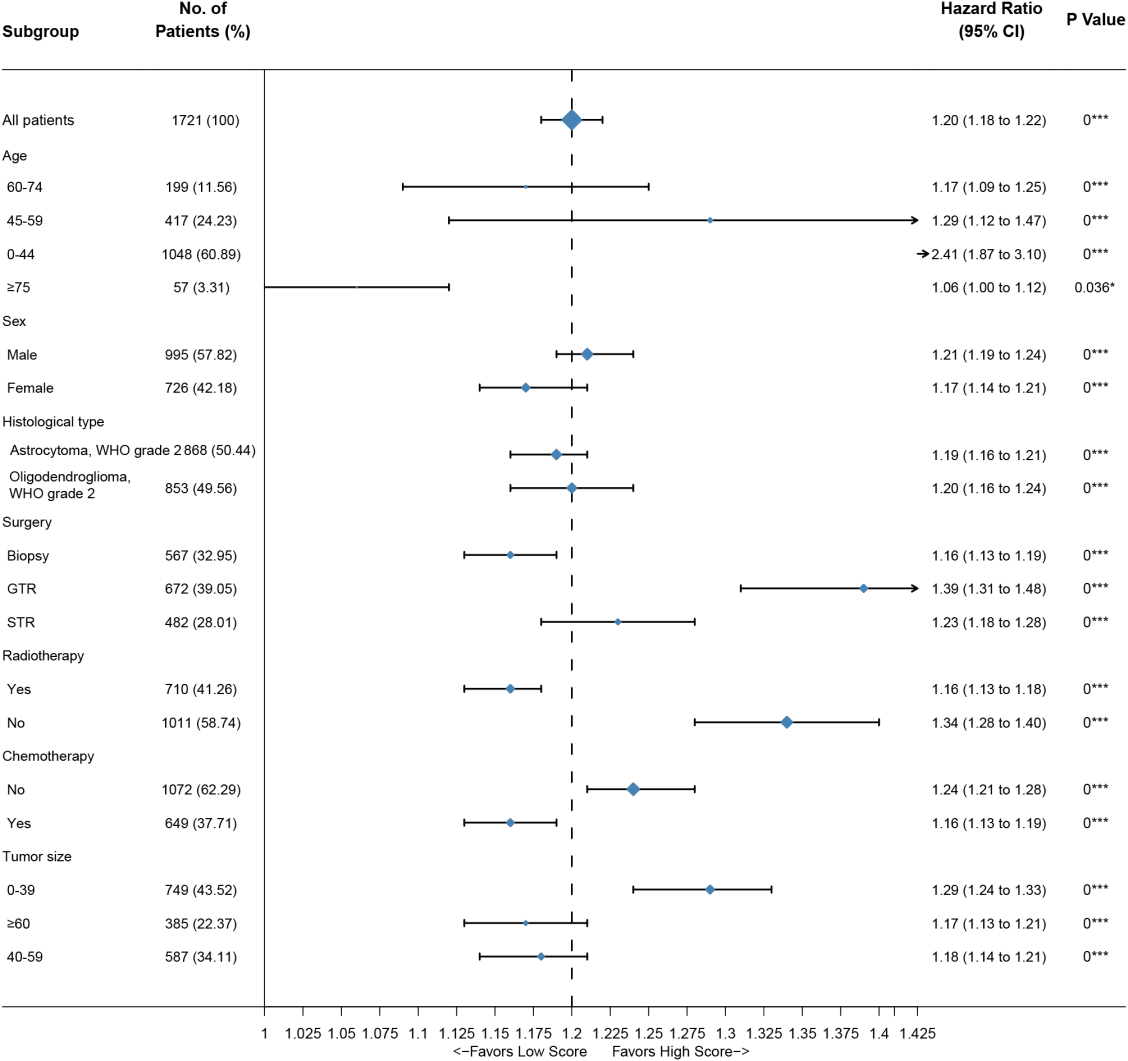
Subgroup analysis of the training group. A straight line with a coordinate of 1 perpendicular to the x-axis is an invalid line. Lines parallel to the x-axis represent the 95% CI for each variable. A line touching the invalid line indicates no statistical significance (*P>0.05*).

**Figure 6:**
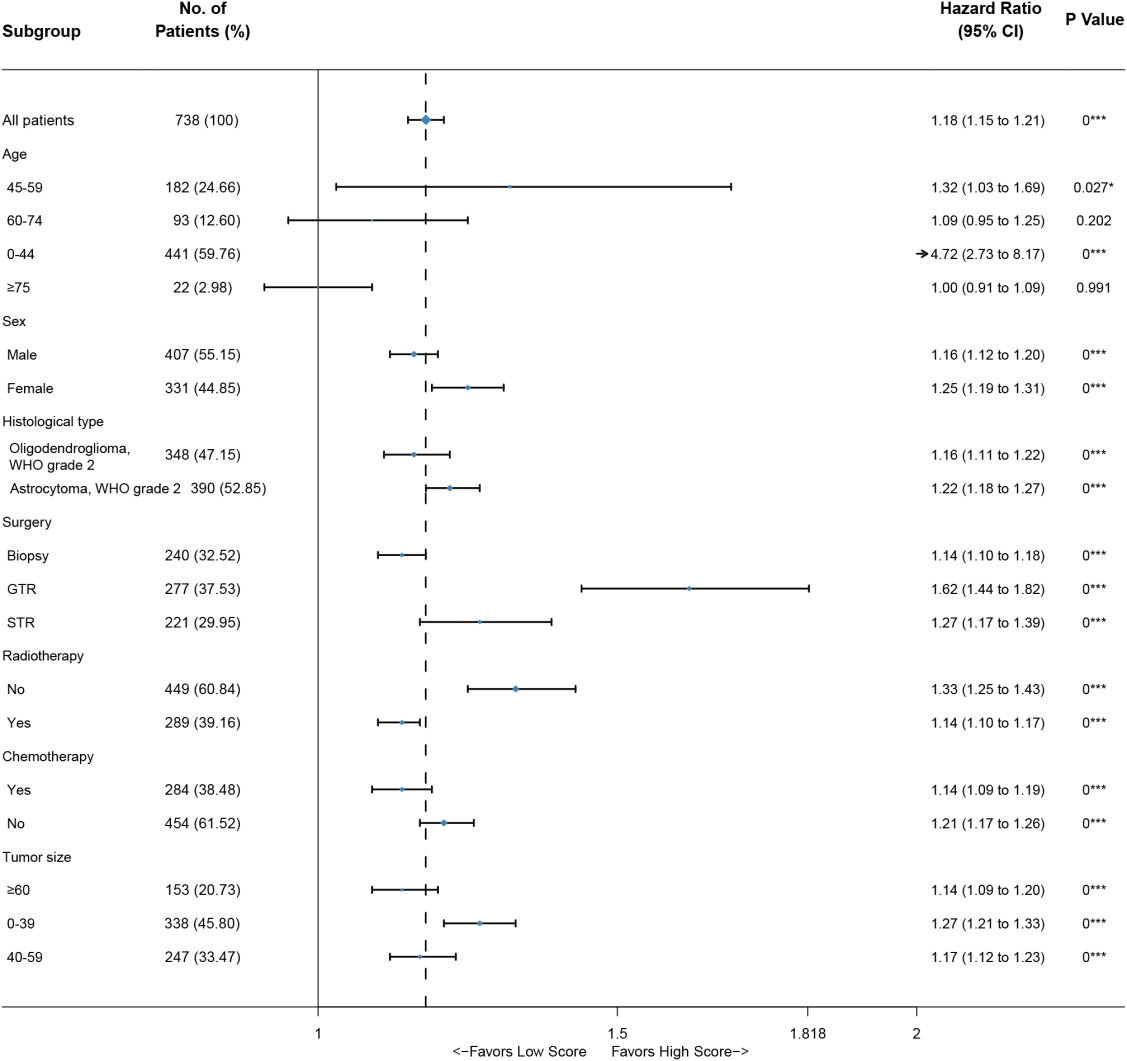
Subgroup analysis of the validation group.

Based on the nomogram, we scored each patient and divided all patients into two groups by the median risk score. As shown in **Figure 4A**, the patients in the high-risk group showed a significantly worse prognosis than those in the low-risk group (*P<0.05*). Then, we divided the DLGG patients into different groups based on their independent prognostic factors to develop a subgroup analysis in the training group (**Figure 5**) and validation group (**Figure 6**). As shown in **Figure 5**, the high-risk group patients always showed a worse prognosis than the low-risk DLGG patients in different age groups (*HR>1, P<0.05*). We also found that the OS of the high-risk group was lower than that of the low-risk group independent of sex (*HR>1, P<0.05*). Regardless of the different tumor resection degrees, the low-risk group always presented a better OS than the high-risk group (*HR>1, P<0.05*). Regardless of whether DLGG patients received radiotherapy (*HR>1, P<0.05*) or chemotherapy (*HR>1, P<0.05*), the high-risk group always had a worse OS than the low-risk group. The high-risk group also had a worse prognosis than the low-risk group regardless of tumor size *(HR>1, P<0.05)*. We found that the OS of the high-risk group was lower than that of the low-risk group regardless of histological type *(HR>1, P<0.05)*. In the training group, subgroup analysis showed that our prediction model was statistically significant for most subgroups (**Figure 5**).

### 3.4 Predictive accuracy of the nomogram

**Figure 7:**
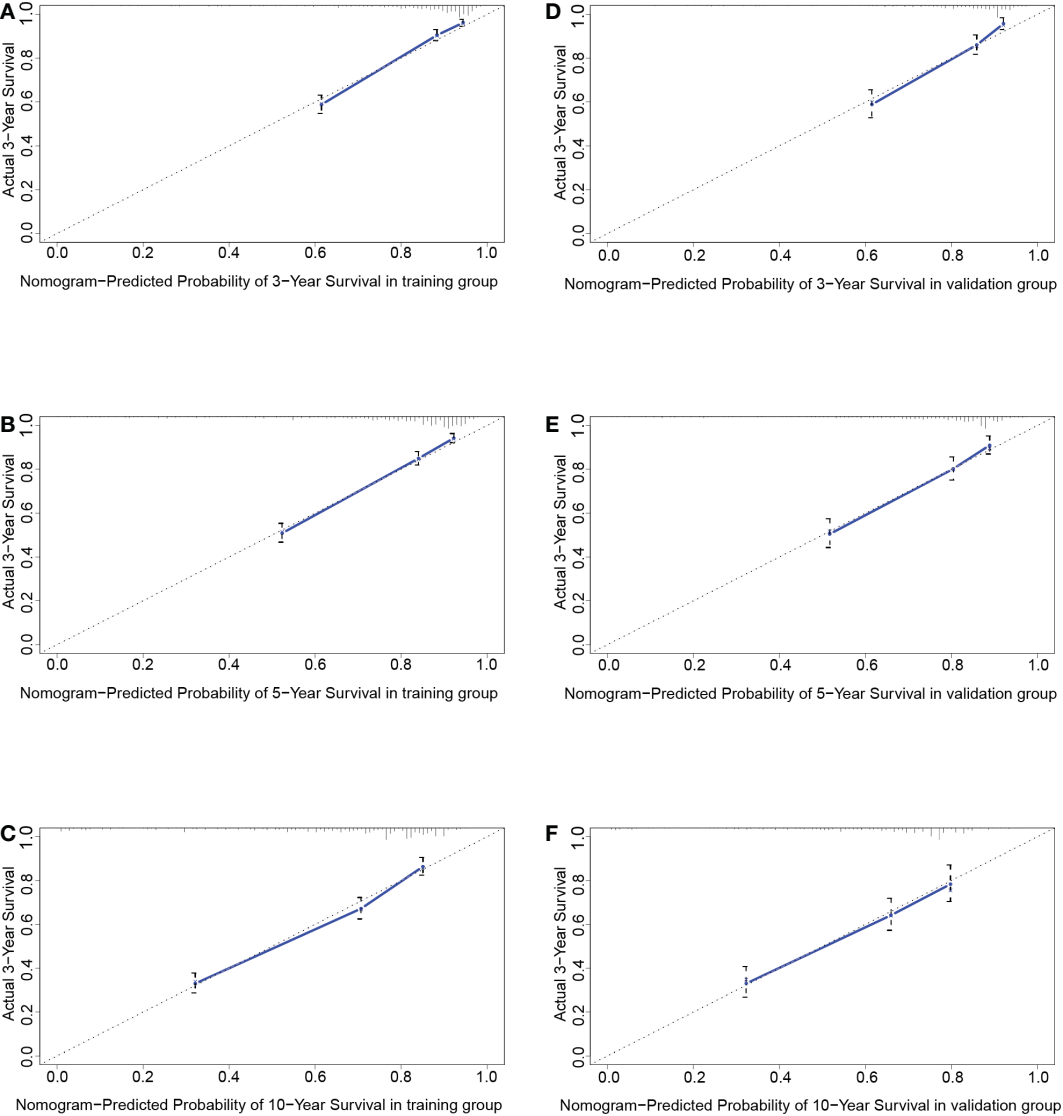
The calibration curves at 3 years **(A)**, 5 years **(B)**, and 10 years **(C)** in the training group and validation group **(D–F)**.

As shown in **Figure 4**, the 3-year, 5-year, and 10-year ROC curves all suggested that the model had good predictive value for DLGG patients [AUCs for 3-year: 0.835, 5-year: 0.815, 10-year: 0.746 (**Figures 4C–E**)]. The C-index of OS prediction in the training group was 0.791. The 3-year, 5-year, and 10-year calibration curves were close to 45° (**Figures 7A–C**). This shows that the nomogram had good predictive value.

### 3.5 External validation

In the validation group, the patients in the high-risk group had a significantly worse prognosis than those in the low-risk group (*P<0.05*) (**Figure 4B**). In the ROC curves of the validation group, the 3-year, 5-year, and 10-year AUCs were 0.818, 0.812, and 0.737, respectively (**Figures 4F–H**). Both the C-index (0.765) and the validation curve showed that the nomogram had a good predictive ability (**Figures 7D–F**). In the age subgroup analysis of validation group, there was no statistical significance for the group of patients aged 60 to 74 years old *(P=0.202, 95% CI: 0.95–1.25, n=93)* and patients older than 75 years old *(P=0.991, 95% CI: 0.91–1.09, n=22)* (**Figure 6**).

## 4 Discussion

For DLGGs, the prognosis varies widely among different patients (7). Here we established a predictive model based on common clinical characteristics to evaluate the prognosis of patients with DLGGs, which is helpful for clinically convenient prognosis evaluation Based on the data from the SEER database, we selected independent prognostic factors and constructed a nomogram, which not only contained the prognostic risk factors but also illustrated the prognosis of each patient (23). In the subgroup analysis, the predictive model also exhibited good performance.

In this study, we found that the independent prognostic factors of the nomogram for the OS of DLGG patients were age, sex, histological type, surgery, radiotherapy, chemotherapy, and tumor size. We found that age had the greatest impact on the OS of DLGG patients. It has been reported that wild-type IDH and malignant tumor biological behavior are more common in older patients with lower-grade gliomas and that higher age is an obvious predictor of a poor prognosis (18, 26–28). In addition, older patients are more likely to suffer from postoperative complications that may change their prognosis (29–31). Multivariate Cox regression analysis showed that the prognosis of patients aged 45-59 (*HR:* 2.5242 *p–value: < 0.05*), 60-74 (*HR:* 5.5916 *p–value: < 0.05*), and ≥ 75 years old (*HR:* 12.6636 *p–value: < 0.05*) was worse than that of patients aged ≤ 44 years old. This further confirms that higher age is a risk factor for DLGG patients.

Epidemiological studies have reported that there are differences in the risk and prognosis of gliomas by sex (32, 33). The survival rate of male patients with glioma is significantly lower than that of female patients (34). Other studies have shown that women with gliomas respond better to surgery, radiotherapy, and standard treatment with TMZ than men (35). This study also concluded that the prognosis of female patients with DLGGs was better than that of male patients (*HR:* 1.1774 *p–value: < 0.05*).

The prognosis of patients with DLGGs is related to the degree of surgical resection (7). Guidelines from The National Comprehensive Cancer Network (NCCN) recommend that tumors in nonfunctional areas should be completely resected (7). Resection of tumors in the functional area should be maximized within a safe range to preserve brain function as much as possible (7, 8). At present, imaging examinations such as MRI and CT can provide a great convenience for surgeons (36, 37). Neuronavigation can be used to locate the tumor and determine the scope of resection during the operation (7, 37). Functional MRI (FMRI) can be used to identify the functional areas of the brain and protect important brain functions such as movement and language (7, 37, 38). In this study, there were significant differences in prognosis among different degrees of tumor resection. The prognosis of patients with gross total resection was better than that of patients with subtotal resection of the tumor, or biopsy (**Figure 3C**).

The results of our study confirmed that postoperative chemotherapy and radiotherapy can improve the OS of patients with DLGGs. The RTOG 0424 trial compared lower-grade glioma patients treated with TMZ alone or with radiation combined with TMZ (RT-TMZ) (39). The results showed that the RT-TMZ regimen had better OS and progression-free survival (PFS) than radiotherapy alone (39). However, for radiotherapy, there are still problems to be solved (40). In a clinical trial, 203 patients with lower-grade gliomas from 1986 to 1994 randomly received low-dose (50.4 Gy) or high-dose (64.8 Gy) radiotherapy after surgery (41). Through long-term follow-up, it was found that compared with low-dose radiotherapy, high-dose radiotherapy did not benefit patients significantly (41). In the future, the best time and dose of radiotherapy for DLGGs deserve further study.

This study showed that tumor size is one of the risk factors for patients with DLGGs. Multivariate Cox regression results showed that the HRs of tumor diameter ≥ 6 cm and 4-6 cm relative to < 4 cm were 1.8198 and 1.4177, respectively. From the Kaplan–Meier survival curve analysis, it can also be concluded that the OS of patients with tumors < 4 cm is the best and that of patients with tumors ≥ 6 cm is the worst.

In this study, not all the results were statistically significant. In the age subgroup analysis of validation group, there was no statistical significance for the group of patients aged 60 to 74 years old *(P=0.202, 95% CI: 0.95–1.25, n=93)* and patients older than 75 years old *(P=0.991, 95% CI: 0.91–1.09, n=22)* (**Figure 6**). By further analysis, we found that the sample size in this subgroup was much smaller than that in other subgroups, which may reduce the statistical power of these results (42). Thus, many more samples are needed to confirm the reliability of these results in the future.

However, this study also has some limitations. First, because the SEER database lacked molecular markers, this study might not reflect the real situation based on the 2021 WHO classification. Second, the only prognostic indicator included in the SEER database was OS and we were unable to predict the prognosis of patients with DLGGs in other aspects, such as PFS and time-to-progression (TTP). Third, the patients identified in the SEER database were mainly white, which would affect the suitability of the model for other races.

In summary, we constructed a clinical predictive model for the prognosis of DLGG patients based on clinical data from the SEER database. Further analysis showed that the model has good prediction performance and reliable results in different clinical subgroups. The results of the validation group also supported this conclusion. Therefore, we believe this model can help predict the survival of DLGG patients.

## 5 Conclusion

The nomogram constructed with common clinical characteristics in this study has good prognostic value for patients with DLGGs. It can help physicians conveniently predict the OS of patients and assist in making clinical decisions.

## Data availability statement

The datasets presented in this study can be found in online repositories. The names of the repository/repositories and accession number(s) can be found below: https://seer.cancer.gov/.

## Author contributions

LA: Conceptualization, methodology, visualization, writing—original draft. DS: Conceptualization, methodology, visualization, writing—original draft. DL: Resources, software. HY: Methodology, writing—original draft. LX: Methodology, writing—original draft. YX: Resources, data curation. SH: Formal analysis. YY: Formal analysis. WZ: Methodology, data curation, writing—original draft. JZ: Methodology, data curation, writing—original draft. HX: Conceptualization, supervision, writing—original draft. All authors contributed to the article and approved the submitted version.
